# The impact of health care strikes on patient mortality: A systematic review and meta‐analysis of observational studies

**DOI:** 10.1111/1475-6773.14022

**Published:** 2022-07-21

**Authors:** Ryan Essex, Sharon Marie Weldon, Trevor Thompson, Erika Kalocsányiová, Paul McCrone, Sanjoy Deb

**Affiliations:** ^1^ Institute for Lifecourse Development University of Greenwich London UK; ^2^ School of Health Sciences University of Greenwich London UK; ^3^ Barts Health NHS Trust, The Royal Hospital London UK; ^4^ Department of Surgery and Cancer Imperial College London, Chelsea and Westminster Hospital London UK; ^5^ School of Human Sciences University of Greenwich London UK; ^6^ School of Life Sciences University of Westminster London UK

**Keywords:** health policy, health systems, health care worker, mortality, protest, strike

## Abstract

**Objective:**

This study sought to evaluate the impact of health care strike action on patient mortality.

**Data Sources:**

EMBASE, PubMed CINAHL, BIOETHICSLINE, EconLit, WEB OF SCIENCE, and grey literature were searched up to December 2021.

**Study Design:**

A systematic review and meta‐analysis were utilized.

**Data Collection/Extraction:**

Random‐effects meta‐analysis was used to compare mortality rate during strike versus pre‐ or post‐strike, with meta‐regression employed to identify factors that might influence the potential impact of strike action. Studies were included if they were observational studies that examined in‐hospital/clinic or population mortality during a strike period compared with a control period where there was no strike action.

**Principal Findings:**

Seventeen studies examined mortality: 14 examined in‐hospital mortality and three examined population mortality. In‐hospital studies represented 768,918 admissions and 7191 deaths during strike action and 1,034,437 admissions and 12,676 deaths during control periods. The pooled relative risk (RR) of in‐hospital mortality did not significantly differ during strike action versus non‐strike periods (RR = 0.91, 95% confidence interval 0.63, 1.31, *p* = 0.598). Meta‐regression also showed that mortality RR was not significantly impacted by country (*p* = 0.98), profession on strike (*p* = 0.32 for multiple professions, *p* = 0.80 for nurses), the duration of the strike (*p* = 0.26), or whether multiple facilities were on strike (*p* = 0.55). Only three studies that examined population mortality met the inclusion criteria; therefore, further analysis was not conducted. However, it is noteworthy that none of these studies reported a significant increase in population mortality attributable to strike action.

**Conclusions:**

Based on the data available, this review did not find any evidence that strike action has any significant impact on in‐hospital patient mortality.

**What is known on this topic**

- Strike action in health care is a contentious issue raising a range of ethical, regulatory, and legal questions.
- While a number of studies suggest strike action has minimal impacts on patient outcomes, this remains disputed.

**What this study adds**

- This review did not find any evidence that strike action had any impact on in‐hospital mortality.
- Country, duration, and the profession on strike had little impact on in‐hospital mortality.
- While only three studies examined population mortality, these found that mortality was not attributable to strike action.

## 1 INTRODUCTION

Strike action when carried out by health care workers has been a particularly contentious issue, with debate and controversy spanning several decades. A strike is distinct from other forms of workplace activism or resistance in that it involves a temporary withdrawal of labor as a means to raise some kind of grievance. Such action has been a frequent occurrence across the globe and has varied in length and scale, with strikes lasting hours to hundreds of days,^1^ impacting local clinics to entire countries.^2^ Perhaps more broadly, the political climate in which strikes have occurred, and the health care systems and patients they have impacted have varied substantially.^3^ While demands have most frequently been about workplace pay and conditions, strike action has been utilized to raise a range of other grievances.^4^

Strikes when undertaken by health care workers raise a range of ethical, regulatory, and legal issues. One issue that weighs heavily in discussions relates to the well‐being of patients, namely the impact that strike action could have on patient care. Arguably the most cited and contentious concern relates to patient mortality.^5^ One does not have to look far to find polarizing debate in the literature, with arguments that point out that “[t]he sick and the wounded are regarded as outside the battlefield even in bitter and bloody conflicts.”^6^ Such concerns have also weighed heavily for professional and regulatory bodies, for example, during the 2016 UK junior doctors strikes, the General Medical Council (GMC; the UK's regulatory body for doctors) issued a stark warning, urging for strike action to be called off, stating that, “we believe that, despite everyone's best efforts, patients will suffer.”^7^

While these debates are likely to persist, the impact of strikes can and has been measured, with a growing literature examining the impact of strike action on health care delivery, health worker attitudes, and importantly, in this case, patient mortality. Given this and given the fact that strike action will continue to be a frequent feature of health care into the foreseeable future,^8^ there is a pressing need for greater clarity in relation to its impact on patient mortality. This study therefore seeks to examine the impact of health care strike action on in‐hospital and population mortality.

## 2 REVIEW QUESTION

What is the impact of health care strike action on patient mortality outcomes globally?

## 3 METHODS

A systematic review was carried out to identify all relevant studies related to patient mortality during strike action. Our approach followed PRISMA guidelines.^9^ Our study protocol was registered on PROSPERO (registration number CRD42021238879), peer reviewed, and published.^10^

### 3.1 Search strategy

A search was conducted on December 17, 2021. The following electronic databases and time periods were searched: EMBASE (1980–2021), MEDLINE (1946–2021), CINAHL (1982–2021), BIOETHICSLINE (1972–1999), EconLit (1969–2021), WEB OF SCIENCE (1960–2021). In addition, grey literature was searched through SIGMA REPOSITORY. While we had planned to search OPEN GREY as per our protocol, it was archived in mid‐2021. Search terms were developed to capture the core concepts related to the form of intervention we were interested in (e.g., strike action, industrial action) and the populations in question (e.g., doctors, nurses, health care professionals).

The final search terms were strike OR “industrial action” OR “industrial dispute” OR “collective action” AND doctor OR physician OR clinician OR “medical practitioner” OR nurs* OR “health profession*” OR healthcare OR “health care” OR “pharmac*” OR “dentist” OR “midwi*” OR dieti* OR “occupational therap*” OR “paramed*” OR “physiotherap*” OR “radiograph*” OR “psycholog*” OR “health worker” OR “hospital.”

There were no publication dates or language restrictions. Where complete data for a relevant outcome was not available, we contacted authors to request data. In addition, we conducted a manual search of reference lists of eligible studies.

### 3.2 Eligibility

We included observational studies (cross‐sectional or cohort studies) that examined in‐hospital/clinic or population mortality during a strike period compared with a control period where there was no strike action. We only included papers where health care professionals went on strike (as opposed to non‐professional health care staff) and where health care services were directly impacted by the strike, not services that dealt with the “upstream” effects of a strike, for example, a hospital where staff were not on strike, but that dealt with excess patients from a nearby hospital on strike (Table 1).

**TABLE 1:** Eligibility criteria

	Inclusion criteria	Exclusion criteria
Population	Patients presenting or admitted to hospital or a health care service (in‐hospital/clinic mortality) and the general/local population (population mortality)	Outpatient services; Alternative health‐related services
Exposure	Period of strike by health care professionals	Strike of non‐professional health care staff or health care services where health care professionals have not gone on strike (e.g., upstream effects of strike)
Comparator	Period of no strike by health care professionals (pre and post)	
Outcomes	Mortality	Morbidity
Study design	Observational studies comparing patient mortality during and pre‐/post‐strike	Qualitative studies and other studies that are not observational such as experimental studies.

### 3.3 Primary outcomes

- In‐hospital/clinic mortality during a strike period as a proportion of admissions was examined against a comparable time period before and/or after the strike action. Mortality rates for each period were compared.
- Population mortality during a strike period was examined against a comparable time period before and/or after the strike action. Mortality rates for each period were compared.

### 3.4 Screening and data extraction

Two authors (RE and SMW) conducted the first screen of titles and abstracts to confirm eligibility. A second screen was then undertaken by RE and SMW, which examined the full text of the remaining articles against the above eligibility criteria. Disagreements were resolved through discussion or with a third member of the review team. For the studies retained, RE extracted study data, which were checked by SMW. Data were extracted related to study characteristics, the nature of the strike, the outcomes of the study, and any other contextual details (Table 2).

**TABLE 2:** Details of included studies

Author	Year	Country	Aims of study	Method/study design	Source of data	Comparison	Study setting	Nature of strike	Number of staff and professions on strike	Length of strike	Hospital or population data	Outcomes of study	GRADE rating
Bhuiyan and Machowski	2012	South Africa	This study sought to examine the effects of the 2010 strike on Polokwane Hospital, comparing performance indicators during the strike with a non‐striking period.	Quantitative–retrospective observational study. Data was collected during the strike period and during a randomly selected 20‐day period in May.	Hospital data from Polokwane Hospital	Pre‐strike	This study says little about Polokwane Hospital; however, note that this hospital, along with surrounding hospital in Limpopo Province, were all rendered “non‐functional”. Only one hospital provided emergency services. About 90% of the 5.5 million people in the province relied solely on public hospitals.	This strike involved doctors and occurred for 20 days (18 August 2010 to 6 September 2010). The authors describe almost all hospitals in the province during the strike as almost “non‐functional”	Doctors (junior doctors)	20 days	Hospital	This study reported that mortality increased as a proportion of overall admissions during the strike.	Very low
Cho	2021	South Korea	This study sought to examine the mortality rate at six training hospital emergency departments during a junior doctors' strike.	Quantitative–retrospective observational study. This study examined the 17 days strike period against two control periods.	Not stated	Pre‐strike	This study occurred across six hospitals in Daegu, South Korea. In 2019, 252,608 patients visited the emergency departments of these hospitals.	This strike occurred in 2020 when junior doctors went on strike. In University and training hospitals, only faculty and senior doctors were available to provide treatment.	Doctors (junior doctors)	17 days	Hospital	This study reported that the strike did not have a significant effect on mortality.	Very low
Daga and Shende	1999	India	This study sought to examine the impact of a strike on mortality and neonatal care during a residents' strike.	Quantitative–retrospective observational study. Data was collected from pre‐strike, strike, and post‐strike periods.	Not stated	Pre and post‐strike	This strike occurred in a rural hospital in India. No other details are provided.	This strike involved doctors and occurred for 69 days, beginning on the 8 November 1991. This study notes that substantial efforts were made to train staff in neonatal care in preparation for the strike.	Doctors (junior doctors)	69 days	Hospital	This study reported no significant difference in mortality compared to pre and post‐strike periods.	Very low
Erceg, M.	2007	Croatia	The study sought to examine whether a doctors' strike in Croatia had an impact on population mortality.	Quantitative–retrospective observational study. Deaths were grouped into three periods (strike, pre, and post). Further comparisons were made over four years, between 2000–2004.	National Bureau of Statistics' deceased people database.	n/a	This study examined deaths across all of Croatia.	This strike occurred in 2003 and lasted for 30 days. The majority of doctors went on strike; however, a number of contingencies were put in place to maintain acute care during the strike.	Doctors	30 days	Population	This study reported no increase in mortality during the strike period or when compared to other non‐strike periods.	Very low
Furnivall et al.	2018	United Kingdom	This study sought to examine the impacts of the four episodes of industrial action by English junior doctors in early 2016.	Quantitative–retrospective observational study. Data was collected from strike periods and compared to comparator weeks, calculated from data extracted from periods immediately preceding and following the strike.	Hospital data from England‐wide NHS Hospital Episode Statistics.	Mean period of time	This study includes data from all hospitals throughout England.	This action occurred in the first 4 months of 2016 when junior doctors from all specialties in England engaged in industrial action with a series of 24–48 h strikes (12 January; 10 February; 9–10 March; and 26–27 April). The final strike included the withdrawal of emergency services. During industrial action, medical outpatient clinics were canceled (other than urgent cancer clinics) and consultants provided ward cover and managed the acute take.	Doctors (junior doctors)	6 days (intermittent)	Hospital	This study reported that mortality did not increase significantly on strike days.	Very low
Gruber and Kleiner	2012	United States	This study sought to analyze the effects of nurses' strikes in hospitals on patient outcomes in New York State over 1984–2004.	Quantitative–retrospective observational study. Hospitals that were on strike were compared to control hospitals in their area. An event‐study approach was used to examine outcomes before, during, and after a strike in the striking versus control hospitals.	Health care union data submitted to the Federal Mediation and Conciliation Service was cross‐referenced with data held by New York State Department of Health	Mean period of time	This study included 43 different hospitals in New York State.	This study examined multiple strikes from 1984 to 2004 period in New York State. The final sample in this study included 50 strikes in 43 hospitals between 1983–2004. Five hospitals were struck twice and one was struck three times. Median strike duration was 19 days, mean duration was 32 days. During 21 of the 50 strikes, hospitals admitted fewer than 30 patients per day.	Nurses	32 days (mean length)	Hospital	This study reported that after controlling for hospital‐specific heterogeneity, nurses' strikes increase in‐hospital mortality by 18.3%.	Low
Harvey et al.	2008	New Zealand	This study sought to utilize a junior doctor strike to examine the hypothesis that increased seniority of emergency department medical staff would result in improved efficiency.	Quantitative–prospective observational study. Data was collected from strike periods and compared with data from non‐strike days (corresponding days of the subsequent calendar week).	Hospital data	Post‐strike	This study was carried out in Waikato Hospital, a 650‐bed hospital located in Hamilton. The hospital provided tertiary‐level care and acts as a trauma center for a local population of 190,000. The emergency department has an annual attendance of 51,000, of which 20% are pediatric. Admission rate is approximately 35%.	This strike involved junior doctors and lasted 5 days (starting on June 15, 2006). All patients who presented to the emergency department were seen by senior doctors.	Doctors (junior doctors)	5 days	Hospital	This study reported that there were no differences in emergency department mortality.	Low
James	1979	United States	This study sought to examine the impact of a doctors' strike on mortality in LA County.	Quantitative–retrospective observational study. Exploring actual versus expected deaths for the first 7 weeks of 1976.	Mortality and population data from the Office of Records and Statistics of the LA County Department of Health Services	n/a	This study examined actual versus expected deaths across LA County.	This strike occurred in 1976 and lasted for about one month. Doctors in LA County went on strike in relation to increasing malpractice costs. While the strike only lasted a month, there were work slow downs and unrest around the strike.	Doctors	30 days (approximately)	Population	This study reported no increase in population mortality during the strike, compared to non‐strike periods.	Very low
Kaguthi et al.	2020	Kenya	This study sought to assess the mortality impact of a 100‐day physician strike which was followed by 151‐day nurses' strike and 20‐day clinical officer strike in Kenya.	Quantitative–retrospective observational study. This study included data from four hospitals, three of which were impacted by strikes. Data was collected from the strike period and compared to data pre‐ and post‐strike.	Hospital data, from the following departments: outpatient, casualty, maternity/neonatal, HIV/ART clinic, surgical and medical wards	Pre and post‐strike	This study included four hospitals. This included three public and one private faith led facility. Staff in the private facility did not go on strike and these data were excluded from our analysis. The hospitals included represent a mix of hospital size and level of specialization. Two were in the capital, Nairobi, while one hospital was 430 km outside the capital.	This strike lasted for a total of 250 days. Doctors (December 2016 to March 2017), nurses (June 2017 to November 2017) and clinical officers (September 2017 to October 2017) went on strike for this period. Some public sector health care facilities remained marginally functional as they were staffed by clinical officers and/or nurses. Many other facilities simply closed during the strike. Neither the doctors' union nor striking government physicians made substantive provisions for delivery of emergency or essential health care services, leaving “private” health care facilities, including faith‐based organizations, the only options for access to health care in the country.	Doctors, Nurses, and clinical officers	250 days (total). 100 days (doctors), 150 days (nurse) and 20 days (Clinical officer)	Hospital	This study reported that during the doctors' strike mortality decreased significantly, while mortality was not impacted by the nurse and clinical officer strike.	Very low
Leskovan et al.	2020	United States	This study sought to explore the impact of a nursing/technical services staff strike.	Quantitative–retrospective observational study. Data was collected from the strike period and compared to data pre‐ and post‐strike.	Hospital data from the “trauma registry”	Pre and post‐strike	This study was carried out in Mercy Health St. Vincent Medical Centre, a trauma center with about 60,000 annual emergency room visits and 2400 trauma admissions per year.	This strike occurred on May 6, 2019 and lasted for 63 days. The authors reported that the hospital was given prior warning regarding this strike and put a number of contingencies in place in response.	Nurse and technical services	63 days	Hospital	This study did not report any significant changes in mortality between pre, post, and strike periods.	Low
Njuguna	2015	Kenya	This study sought to examine the impact of a health worker strike in Mombasa County, Kenya.	Quantitative–retrospective observational study. Data were collected from the strike period and compared to a monthly mean.	Hospital data from the Kenya Health Information System	Pre‐strike	This study was carried out in Mombasa County Referral hospital, the largest hospital in Mombasa County. The authors reported that the population of the county was a little over 900,000 and 37% of the population lived in poverty.	This strike occurred in August 2014 when health workers in government facilities in Mombasa County went on strike.	Health workers (doctors, nurses, clinical officers, pharmacists, laboratory personnel, nutritionists) radiologists, and physiotherapists, among others.	14 days	Hospital	This study reported an overall decline in mortality by 37.5%; however, during the strike attendance also decreased by 67.2%, which meant that mortality increased as a proportion of admissions during strike. This study only presents descriptive statistics.	Very low
Ong'ayo, Gerald	2019	Kenya	This study sought to examine the effect on mortality of six strikes by health workers that occurred from 2010 to 2016 in Kilifi, Kenya.	Quantitative‐Population based cohort study. This study examined mortality during strike periods and in the 2 weeks immediately after strikes.	Mortality data from the Kilifi Health and Demographic Surveillance System	Mean period of time	This study examines mortality as it relates to six strikes by health care workers in Kenya. Kilifi county comprised of both rural and semiurban regions in coastal Kenya, with a population of 300,000.	This study examined six strikes that occurred between 2011–2013 in Kilifi county, Kenya. Strikes were carried out by Doctors and Nurses and lasted between 15 and 42 days and lasted 128 days cumulatively.	Multiple	18.5 days (mean for 6 strikes, 128 strike days total)	Hospital	This study reported that the strikes were not associated with obvious changes in overall mortality in Kilifi.	Low
Ruiz et al.	2013	United Kingdom	This study sought to examine the effect of a 24‐h doctors' strike on June 21, 2012 on hospital activity in English NHS hospitals.	Quantitative–retrospective observational study. Data was collected from the strike period and compared to mean mortality over a 3‐week period.	Hospital data from England‐wide NHS Hospital Episode Statistics.	Mean period of time	This study includes data from all hospitals throughout England.	This strike occurred on June 21, 2012 and was in response to proposed pension reforms. Participation in the strike varied. In the London area, >90% of all hospitals worked normally (approximately 10% of planned operations were postponed) and 83% of GP practices were said to have worked normally, with the remaining 17% open but only dealing with urgent cases. A number of non‐emergency cases and outpatient appointments were affected.	Doctors	1 day	Hospital	There were no significant differences number of in‐hospital deaths between the strike and the non‐strike period.	Very low
Salazar et al.	2001	Spain	This study sought to evaluate the indicators of activity and quality within the emergency department during a junior doctor strike.	Quantitative–retrospective observational study. Data were collected from the strike period and compared to a non‐strike period, matched by weekday.	Hospital information system	Mean period of time	The study was carried out in the Bellvitge Hospital, a 1000‐bed hospital for adults in Barcelona, Spain. The mean number of emergency visits each month is about 9000, with about 300 visits per day.	This strike occurred between May and June 1999. All junior doctors (with the exception of family medicine doctors) participated in this strike for nine non‐consecutive days. More experienced doctors staffed the emergency department during these periods.	Doctors (junior doctors)	9 days	Hospital	This study reported that there was no statistically significant difference in mortality rated between strike and non‐strike periods.	Very low
Sim	2021	South Korea	This study sought to explore the impact of a nationwide strike on emergency department performance.	Quantitative–retrospective observational study. This study examined mortality during a strike period (17 days) and a corresponding control period prior to the strike.	“Hospital information system”	Pre‐strike	This study was conducted in a tertiary‐care academic hospital with 997 inpatient beds. The emergency department served approximately 32,000 patients annually.	This strike occurred in 2020 when junior doctors went on strike. In University and training hospitals, only faculty and senior doctors were available to provide treatment.	Doctors (junior doctors)	17 days	Hospital	This study reported that the strike did not have a significant effect on mortality.	Low
Slater and Ever‐Hadani	1983	Israel	This study sought to examine population mortality during a doctors strike in Israel.	Quantitative–retrospective observational study. Data was collected from around the strike period in 1983 and corresponding control period in 1982.	Death certificates filed in the Jerusalem District Health Office.	Pre‐strike	At the time of the study Jerusalem had a population of 340,000 and was served by four hospitals and 28 clinics. During the strike seven impromptu aid stations functioned in the city.	This strike occurred in 1983 and lasted 118 days. Eight thousand salaried physicians walked off the job on 2 March 1983 and did not return to work until 27 June, when their “salary and status” dispute was submitted to binding arbitration.	Doctors	118 days	Population	This study reported no difference in deaths between the two years throughout the strike.	Very low
Youssef	2021	United States	This study sought to examine the impact of a nursing strike on emergency department metrics.	Quantitative–retrospective observational study. This study examined mortality during a strike period (5 days) and a corresponding control period prior to the strike.	The departments electronic tracking and charting system.	Pre‐strike	This study occurred in a Boston emergency department, which dealt with trauma and saw approximately 46,000 patients annually.	This strike occurred in 2017 when nursing staff in a teaching hospital in Boston went on strike. Replacement nurses were hired by the hospital to fulfill nursing duties for 5 days.	Nurses	5 days	Hospital	This study reported no significant changes in mortality when comparing strike to non‐strike periods.	Low

### 3.5 GRADE assessment and quality appraisal

To assess the certainty of the evidence, the GRADE approach^11^ was applied. This approach specifies four levels of certainty for a body of evidence for a given outcome: high, moderate, low, and very low. Studies are assessed against five criteria: risk of bias, imprecision, inconsistency, indirectness, and publication bias. Observational studies start with a “low” rating, and this can be either increased or decreased against the above criteria. Additionally, study quality was assessed using the NIH quality assessment tool^12^ for observational cohort and cross‐sectional studies and the Cochrane Risk Of Bias In Non‐Randomized Studies‐of Interventions (ROBINS‐I) tool,^13^ which rates the potential for study bias arising pre‐intervention (confounders, participant selection), during the intervention (classification of intervention), and after intervention (deviations, missing data, outcome measurement, result selection). The quality of the study evidence was evaluated by two authors (EK and RE) and two authors (SD and SMW) examined a random sample of assessments.

### 3.6 Publication bias

Potential publication bias was examined visually with funnel plots of effect sizes against SEs (where asymmetry can indicate possible bias), and statistically with Egger's test^14^ with *p* < 0.10 indicating possible publication bias.

### 3.7 Analysis

Meta‐analysis was used to systematically synthesize the findings of the single, independent studies retrieved from the search and included for analysis. The relative risk (RR) was calculated for each study. We pooled RRs using a random‐effects model and tested for heterogeneity. RevMan^15^ and the metafor package in R^16^ were used to carry out the analyses.

### 3.8 Heterogeneity

We tested for the existence of heterogeneity with Cochran's *Q* statistic (where *p* < 0.05 indicates heterogeneity is present). We assessed the magnitude of the variation in effect sizes across studies with Higgin's *I* ^2^ statistic, which estimates the proportion of variance in effect sizes due to true heterogeneity (from 0% to 100%), with higher values representing greater inconsistency in effect size across studies. We also report *τ* as a measure of heterogeneity for each comparison, which gives the SD of the effect size estimates. If heterogeneity was high (*I* ^2^ > 75%), we planned to conduct a meta‐regression to explain possible sources of variation, examining the following potential moderators: strike duration (days); country (low‐ and middle‐income country vs. high‐income country); profession on strike (e.g., doctors, nurses, multiple roles), and whether the strike involved a single or multiple facilities. These were chosen as they were identified as being the main differential factors that were consistently reported between studies.

## 4 RESULTS

**FIGURE 1:**
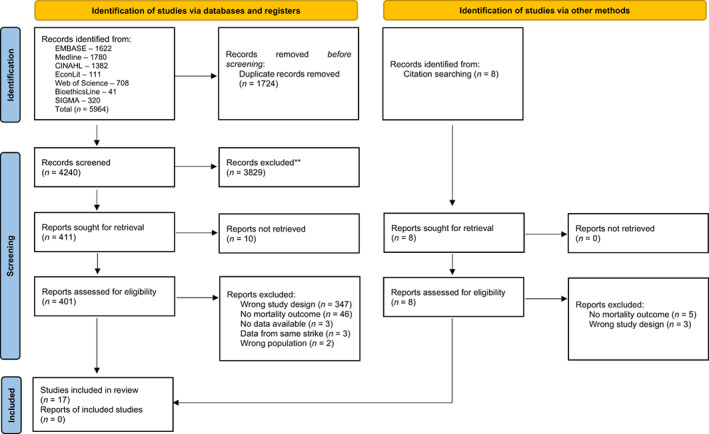
PRISMA 2020 flow diagram [Color figure can be viewed at wileyonlinelibrary.com]

The search returned 5964 results. These were imported into Endnote where duplicates were removed, leaving 4240 articles. After the initial abstract screen (carried out by RE and SMW), 411 articles remained and a second detailed screen was undertaken and reference lists were searched. A further four papers were found, and all 415 articles were assessed against the above eligibility criteria (by RE and SMW), leaving 17 papers that examined mortality^17^, ^18^, ^19^, ^20^, ^21^, ^22^, ^23^, ^24^, ^25^, ^26^, ^27^, ^28^, ^29^, ^30^, ^31^, ^32^, ^33^ (Figure 1). Of these papers, 14 examined in‐hospital mortality, while three examined population mortality. In terms of the studies that examined in‐hospital mortality, five studies used a comparison period pre‐strike, one used a comparison post‐strike, three used comparison periods pre‐ and post‐strike action, while the remaining five studies compared admissions and mortality to a period of time that was not immediately pre‐ or post‐strike, for example, mean rates of admissions and deaths over a specified period were often calculated and used as a comparator for the strike period. All admissions, whether collected pre, post, or at another point in time, were summed to give total admissions and mortality.

Overall, the studies included that examined in‐hospital/clinic mortality represent 768,918 admissions and 7191 deaths during strike action globally and 1,034,437 admissions and 12,676 deaths during comparison periods. Studies were conducted from 1979 to as recently as 2021 (during the COVID‐19 pandemic). The majority of studies (*n* = 11) were conducted in the last decade. Almost every continent was represented with four studies from the United States, three from Kenya, two from South Korea and the United Kingdom, with the remaining studies conducted in Croatia, India, Israel, New Zealand, South Africa, and Spain. Across studies, the setting in which the strike occurred varied greatly, from small rural hospitals^19^ to studies that included nationwide data.^21^ The nature of the strike action also varied substantially; however, there was often little detail provided on the number of staff on strike or the contingencies put in place in regards to patient care. Eleven of the strikes were carried out by doctors, with seven of these being junior doctor strikes. There were three nursing strikes and three strikes where multiple professional staff (e.g., doctors and nurses) went on strike. The length of the strikes ranged from 1 to 250 days; the mean length of strike action was 41 days, while the median length was 18 days.

### 4.1 Risk of bias and study quality

Overall, the certainty of the evidence was “very low” when applying the GRADE approach. Studies mainly had issues on domains related to risk of bias and inconsistency. That is, the majority of studies had at least some limitations in their execution, not including important details about the strike or patient cohorts, heterogeneity was also high. Given the lack of information often found in studies, indirectness was also found to be an issue. The GRADE ratings for each study are included in Table 2, and a GRADE evidence profile is included in Appendix Table 4. Results of the Cochrane risk of bias assessments (Appendix Figures 3 and 4) present an overall unclear risk of bias, with the measurement of outcomes the only domain where bias could be considered low. Higher risk of bias was reported in relation to confounding, selection of participants into the study, missing data, and in selection of the reported results. The NIH quality assessment tool for observational cohort and cross‐sectional studies painted a similar picture (Appendix Figure 5), with the results painting an unclear picture in relation to the quality of studies. Substantial problems were identified in relation to items 9, 10, 12, and 14 (exposure measures, how frequently the exposure was assessed, blinding of assessors, and adjustment for confounders), with the majority of studies failing to address these issues. A substantial number of criteria were rated as “not applicable,” “not reported,” or “could not determine,” with items 3–6 (participation rate, recruitment from similar populations, sample size justifications, and measurement of exposures of interested) and 13 (follow‐up after baseline) all with a substantial number of studies that scored in these categories. Relative strengths related to items 1, 7, and 11 (having a clear research question, sufficient timeframes for exposure and outcomes, whether the outcomes measures were clearly defined and implemented) with the majority of studies addressing these criteria. No evidence of potential publication bias was found, based on the funnel plots (Appendix Figure 6) and a nonsignificant Eggers test (*p* = 0.74).

### 4.2 In‐hospital mortality

**FIGURE 2:**
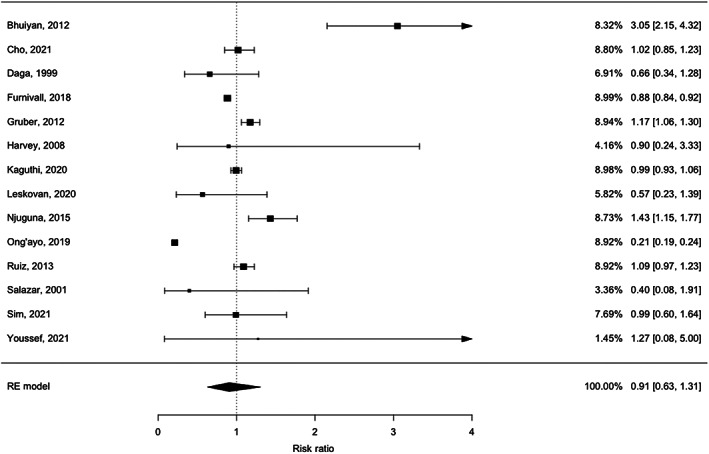
Forest plot of risk‐ratios on the impact of strike action on in‐hospital mortality

The pooled relative risk of mortality during strike action versus not during strike action was not significant (RR = 0.91, 95% confidence interval 0.63–1.31, *p* = 0.598) (Figure 2). Because significant (*Q* = 655.3, *p* < 0.001) and substantial heterogeneity (*I* ^2^ = 98%, *τ* ^2^ = 0.38) were also observed, we conducted a meta‐regression to try to identify sources that might explain this heterogeneity. Results indicated that mortality was not significantly impacted by country (RR = 0.99, *p* = 0.98), profession on strike (RR = 0.63, *p* = 0.32 for multiple professions, RR = 0.87, *p* = 0.80 for nurses), the duration of the strike (RR = 0.99, *p* = 0.26), or when multiple facilities were on strike (RR = 0.71, *p* = 0.55). Also see Table 3 for more information.

**TABLE 3:** Meta‐regression analysis examining whether differences in mortality across strike versus non‐strike periods were moderated by length of strike, country economy, profession, and number of facilities on strike

	Number of studies	Covariates	Regression coefficient	95% CI	*p* Value
Country	14	LMIC^a^	0.99	0.45–2.13	0.980
Length of strike	14	Length of strike	0.99	0.99–1.00	0.268
Staff on strike	14	Multiple professions^b^	0.63	0.26–1.53	0.317
		Nurses^b^	0.87	0.30–2.48	0.795
Site	13	Multisite^c^	0.71	0.23–2.13	0.546

Abbreviations: CI, confidence interval; LMICs, low‐ and middle‐income countries.

^a^ Reference group = High‐income countries.

^b^ Reference group = Doctors.

^c^ Reference group = Single site.

### 4.3 Sensitivity analysis

A sensitivity analysis was conducted with Bhuiyan and Machowski^17^ removed as this study was of particularly low quality and a clear outlier. As its exclusion had no impact on the overall results, we opted to retain this study.

### 4.4 Population mortality

While this review also sought to analyze population mortality related to strike action, further analysis was not possible or appropriate. From the above search, five papers were found that contained population mortality data; however, only three were potentially suitable for analysis. Three papers reported data from the same strike that impacted Los Angeles county in 1976,^24^, ^34^, ^35^ one reported data from a strike that impacted Jerusalem in 1983^32^ and one reported data from a nationwide strike in Croatia in 2003.^20^ Given that three studies examined the same strike, it only left three studies that could be included in any potential analyses. While further analysis was not possible, it is perhaps noteworthy that none of the above studies reported a significant increase in population mortality that was attributable to strike action.

## 5 DISCUSSION

Based on data that were available to us, this review did not find any evidence that strike action has a significant impact on in‐hospital patient mortality. Furthermore, the impact of strike action does not appear to be affected by country, duration, or profession on strike. While we were unable to analyze the impact of strike action on population mortality, the small number of studies that were found did not report any significant increase in population mortality that could be attributed to strike action.

Caution, however, is warranted in interpreting these results. Firstly, the above studies overall were of a relatively low quality scoring poorly on all three quality appraisal instruments. This means that we can only have minimal confidence in the results reported above. There could of course also be alternative explanations as to why strike action did not impact mortality that we also cannot rule out; namely, many strike‐impacted hospitals were able to put contingency plans in place, minimizing the disruption and impact on patient mortality. While several studies provided detailed accounts of the contingencies put in place during strike action to minimize disruption, many did not. Secondly, and related to these points, the nature of the strikes reported varied substantially, as did the health care systems they impacted. In many papers little detail was included about the nature of the strike or the context in which it was occurring, for example, the number of staff on strike or how well resourced the health care system was to cope with the action. While we have included four variables (country, length, staff on strike, and whether the strike occurred in a single or multiple facilities), these should not be seen to capture all nuances and features of a strike. Furthermore, because of the lack of information included in studies, it was often not clear how or if these factors contributed to any disruption, for example, even strikes that we might intuitively expect to cause the most disruptive (for example, those that were protracted and involved multiple facilities), actually did not, with a number of studies suggesting this was actually not the case. Ruiz^29^ for example examined a nationwide strike in the United Kingdom where >90% of hospitals functioned as normal. Thirdly, while this review focused on mortality, it would be insightful to assess the impact of strike action on health‐related quality of life and patient satisfaction with care. Fourthly, a number of studies also failed to report patient characteristics during strike and control periods, that is, most studies have little information on those who sought care during a strike versus those who sought care in non‐strike periods; we therefore cannot be sure if or how this impacted the above results. Finally, there are several studies examining the upstream impacts of strike action (that were found in this search but subsequently excluded) that suggest that strike action had a significant impact on presentations and mortality.^36^ It is possible that while presentations and mortality decreased in strike‐impacted hospitals, many sought treatment elsewhere.

Following this, there are several implications for future research. Firstly, there is a need for future studies that examine strike action to include greater detail about the cohorts being examined and the nature of the strike. There also needs to be a broader examination of factors beyond in‐hospital mortality, linking these data with population statistics about mortality. We also found few studies that linked mortality and other patient outcomes. Secondly, there is also a need to better understand how strike action changes access to care. The limited literature that does exist suggests that generally, while many will delay seeking care, others will seek care elsewhere.^35^ Future studies will benefit from integrating these insights alongside mortality data to understand how strike action impacts mortality in strike‐impacted facilities and those dealing with its upstream effects.

While our results say little about the impact that strike action has on health care delivery, our results are consistent with a broader body of work that suggests that strike action has minimal impact on a range of other patient outcomes,^37^ and past work on the impact of strike action on mortality.^38^ At a minimum, this review suggests that strike action by health care workers can be conducted safely as it relates to patient mortality. This has several implications for debates in relation to the impact of strike action, its justification, and the other legal and regulatory concerns that such action raises. Most notably, while patient outcomes are a valid concern that should weigh heavily in discussions about the justifiability of strike action, strike action should not be dismissed on this point alone as it is far from inevitable that patients will be harmed when health care workers go on strike.

## Supporting information


**Data S1.** Supporting figures and tables.

**Appendix S1.** Search strategy.
