# Supplementary material for: The impact of health care strikes on patient mortality: A systematic review and meta‐analysis of observational studies
Source: Health Serv Res. 2022 Jul 21;57(6):1218–34. doi: 10.1111/1475-6773.14022 (PMC9643090; doi:10.1111/1475-6773.14022)

# Appendix/Supplementary material – Tables and figures

**Table 4. Grade Evidence Profile**

**Strike compared to non-strike periods on in-hospital mortality**

PECOS: Population - patients presenting or admitted to hospital or a healthcare service (in hospital/clinic mortality); Exposure - period of strike by healthcare professionals; Comparator - period of no strike by healthcare professionals (pre and post); Outcome -mortality; Study design - observational studies comparing patient mortality

| **Quality assessment** | | | | | | | **Summary of findings** | | | | **Importance** |
| --- | --- | --- | --- | --- | --- | --- | --- | --- | --- | --- | --- |
|  |  |  |  |  |  |  | **No of patients** | | **Effect** | **Quality** |  |
| **No of studies** | **Design** | **Limitations** | **Inconsistency** | **Indirectness** | **Imprecision** | **Other considerations** | **Intervention** | **Control** | **Relative (95% CI)** |  |  |
| 14 studies (1,803,355 patients total) | Observational studies | Serious issues | Serious issues | Some issues | None | None | 768,918 | 1,034,437 | RR 0.91 (0.63, 1.31) | ⊕🌕🌕🌕  VERY LOW | Critical |

**Figure 3. ROBINS I results by study**


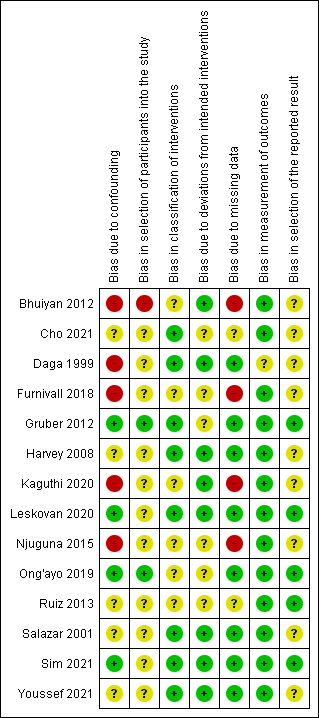


Note: Only studies included in the in-hospital analysis were rated

**Figure 4. ROBINS I results summary**


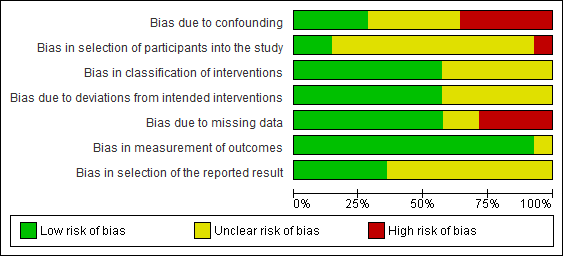


Note: Only studies included in the in-hospital analysis were rated

**Figure 5. NIH results summary**

Note: Only studies included in the in-hospital analysis were rated

**Figure 6. Funnel plots of effect sizes against standard errors**


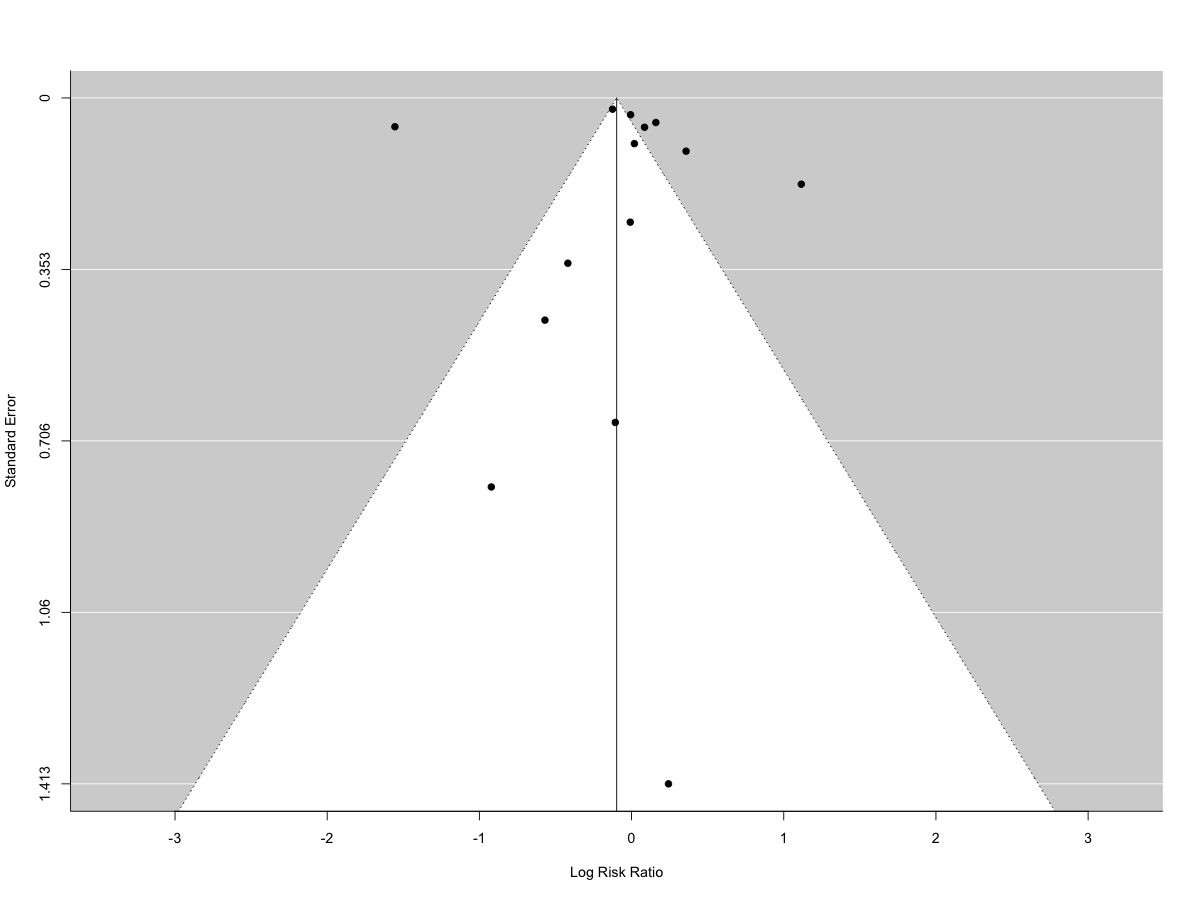

Supplement: Supplementary file 1 — Data S1. Supporting figures and tables. [file HESR-57-1218-s001.docx]
