# Supplementary material for: The impact of health care strikes on patient mortality: A systematic review and meta‐analysis of observational studies
Source: Health Serv Res. 2022 Jul 21;57(6):1218–34. doi: 10.1111/1475-6773.14022 (PMC9643090; doi:10.1111/1475-6773.14022)
Supplement: Supplementary file 2 — Appendix S1. Search strategy. [file HESR-57-1218-s002.docx]

**Appendix 1. Search strategy**

EMBASE

((strike or "industrial action" or "industrial dispute" or "collective action") and (doctor or physician or clinician or "medical practitioner" or nurs* or "health profession*" or healthcare or "health care" or pharmac* or dentist or midwi* or dieti* or "occupational therap*" or paramed* or physiotherap* or radiograph* or psycholog* or "health worker" or hospital)).ab

MEDLINE

((strike or "industrial action" or "industrial dispute" or "collective action") and (doctor or physician or clinician or "medical practitioner" or nurs* or "health profession*" or healthcare or "health care" or pharmac* or dentist or midwi* or dieti* or "occupational therap*" or paramed* or physiotherap* or radiograph* or psycholog* or "health worker" or hospital)).ab.

CINAHL

AB ( strike or "industrial action" or "industrial dispute" or "collective action" ) AND AB ( doctor or physician or clinician or "medical practitioner" or nurs* or "health profession*" or healthcare or "health care" or pharmac* or dentist or midwi* or dieti* or "occupational therap*" or paramed* or physiotherap* or radiograph* or psycholog* or "health worker" or hospital )

EconLit

AB ( strike or "industrial action" or "industrial dispute" or "collective action" ) AND AB ( doctor or physician or clinician or "medical practitioner" or nurs* or "health profession*" or healthcare or "health care" or pharmac* or dentist or midwi* or dieti* or "occupational therap*" or paramed* or physiotherap* or radiograph* or psycholog* or "health worker" or hospital )

WEB OF SCIENCE

TITLE: (strike or "industrial action" or "industrial dispute" or "collective action") AND TITLE: (doctor or physician or clinician or "medical practitioner" or nurs* or "health profession*" or healthcare or "health care" or pharmac* or dentist or midwi* or dieti* or "occupational therap*" or paramed* or physiotherap* or radiograph* or psycholog* or "health worker" or hospital)

BIOETHICSLINE

(strike OR "industrial action" OR "industrial dispute" OR "collective action") AND (doctor OR physician OR clinician OR "medical practitioner" OR nurs* OR "health profession*" OR healthcare OR "health care" OR pharmac* OR dentist OR midwi* OR dieti* OR "occupational therap*" OR paramed* OR physiotherap* OR radiograph* OR psycholog* OR "health worker" OR hospital)

SIGMA REPOSITORY

(strike OR "industrial action" OR "industrial dispute" OR "collective action") AND (doctor OR physician OR clinician OR "medical practitioner" OR nurs* OR "health profession*" OR healthcare OR "health care" OR pharmac* OR dentist OR midwi* OR dieti* OR "occupational therap*" OR paramed* OR physiotherap* OR radiograph* OR psycholog* OR "health worker" OR hospital)
